# Effects of an Eye-Tracking Digital Serious Game on Cognitive Function in Mild Cognitive Impairment: Pilot Intervention Study

**DOI:** 10.2196/88924

**Published:** 2026-05-19

**Authors:** Sang-Woo Lee, Jun-Su Kim, Seung-Jae Kim, Seungho Choun, Jeong-Heon Song

**Affiliations:** 1 InTheTech Inc. Daegu Republic of Korea; 2 Korea Brain Research Institute Daegu, Daegu Republic of Korea

**Keywords:** digital cognitive training, eye tracking, mild cognitive impairment, neurocognitive intervention, oculomotor function

## Abstract

**Background:**

Cognitive decline in aging populations underscores the need for early interventions in mild cognitive impairment (MCI), where pharmacological treatments show limited benefit. Eye-movement metrics serve as sensitive markers of cognitive deficits in MCI, and digital programs integrating these tasks offer scalable, data-driven training approaches.

**Objective:**

This study aimed to evaluate the effectiveness of a digital cognitive training program incorporating eye-movement tasks in individuals with MCI, and to determine whether eye-movement indicators can serve as objective markers of cognitive improvement.

**Methods:**

A total of 12 participants aged 60-85 years with MCI (Korean version of the Montreal Cognitive Assessment [K-MoCA] score of ≤22) completed baseline and postintervention assessments using the K-MoCA and Mini-Mental State Examination-Korean version (MMSE-K). Longitudinal changes in visuospatial attention and oculomotor performance were examined using Spearman correlations across sessions, and pre-post comparisons of eye-tracking metrics were conducted to assess training-related improvements.

**Results:**

Cognitive scores improved significantly, with K-MoCA increasing by 1.5 points (from mean 20.3, SD 1.1 to mean 21.8, SD 1.7; *P*=*.*004; Cohen *d*=1.38) and MMSE-K by 1.3 points (from mean 21.9, SD 2.0 to mean 23.2, SD 2.2; *P*=*.*002; Cohen *d*=1.29). Fixation duration decreased (*r*=0.248; *P*=*.*003), and saccade velocity increased (*r*=0.258; *P*=*.*002), indicating enhanced visual processing efficiency and faster attentional shifts, whereas fixation count and saccade amplitude showed no consistent changes. In addition, saccade duration decreased by 21.72 ms, and saccade velocity increased by 114.54 °/s.

**Conclusions:**

Digital cognitive training yielded measurable gains in visuospatial attention and oculomotor efficiency in MCI, with optimized fixation and saccade patterns indicating enhanced attentional control and information processing. These findings support eye-movement metrics as sensitive indicators of cognitive change and highlight digital interventions as scalable, noninvasive tools for cognitive support in aging populations.

## Introduction

With the rapid global aging of the population, cognitive impairment and dementia have become pressing social and public health concerns. Mild cognitive impairment (MCI) represents a transitional stage between normal aging and dementia [1], characterized by noticeable deficits in memory, attention, and executive function, with largely preserved activities of daily living [2]. Studies report that approximately 8%-15% of individuals with MCI progress to dementia annually, a rate significantly higher than that of cognitively healthy older adults [3]. Consequently, early intervention during the MCI stage is crucial for preventing or delaying the onset of dementia and has become a key focus of contemporary clinical research.

Pharmacological treatments have been primarily developed for dementia, particularly Alzheimer disease, but have demonstrated limited effectiveness in individuals with MCI [4,5]. Moreover, medications are often associated with adverse effects and adherence issues, thereby complicating long-term management [6,7]. Consequently, nonpharmacological interventions, especially cognitive training programs, have emerged as promising alternatives. Traditional cognitive training for MCI typically involves memory enhancement, problem-solving, and memory encoding tasks [5,8,9]. These programs aim to strengthen specific cognitive domains through repetitive practice and have been reported to improve attention, memory, and executive functions [9-12]. For example, group-based training appears to enhance memory and subjective cognitive performance more effectively than individual training [13], whereas attention-focused tasks improve processing speed [14]. Collectively, these findings suggest that cognitive training may slow cognitive decline and improve quality of life [15,16].

Eye movements have recently emerged as important neurophysiological indicators closely linked to cognitive processes. Gaze behavior is task dependent and reflects the underlying perceptual, cognitive, and motor processes associated with selecting and processing relevant sensory information [17]. Individuals with MCI generally exhibit reduced fixation stability, increased unnecessary gaze shifts, as well as slower and less accurate saccadic movements compared with healthy older adults [18]. These changes are often accompanied by deficits in memory, attention, and visuospatial processing, making eye-movement metrics a sensitive tool for detecting cognitive vulnerability in MCI [19]. Furthermore, emerging evidence indicates that eye movement–based tasks improve attention and working memory, suggesting that incorporating eye movements into cognitive interventions may substantially enhance outcomes [18,20].

More recently, traditional cognitive training has increasingly been integrated with digital technology. Digital cognitive interventions offer several advantages, including standardized and personalized training, engaging and interactive environments that encourage sustained participation, and precise collection of response data for quantitative analysis of cognitive change [21]. When eye movement is integrated into such digital programs, fixation and saccade data can provide real-time evaluation of attention, reaction speed, and visual search strategies, enhancing intervention effectiveness [22]. Consistently, some studies have reported that digital cognitive training is more effective than traditional methods in improving memory and executive functions [23,24].

Building on this background, we aimed to rigorously evaluate the effectiveness of a digital cognitive training program integrating eye movement–based tasks in individuals with MCI. Considering the eye-movement patterns often observed in MCI, we hypothesized that digital interventions could yield positive effects across multiple cognitive domains, including attention, memory, and executive function. Ultimately, in this study, we aimed to provide objective evidence supporting the clinical use of digital cognitive training in this population.

## Methods

### Participants

We recruited older adults aged 60-85 years diagnosed with MCI. Eligibility was determined through a score of ≤22 on the Korean version of the Montreal Cognitive Assessment (K-MoCA). A total of 12 participants were enrolled, comprising 5 (41.7%) male and 7 (58.3%) female individuals (Table 1).

**Table 1:** Summary statistics. Of the 12 total participants, 41.7% (n=5) were male and 58.3% (n=7) were female individuals.

Characteristic	Mean (SD)	Median (IQR)	Minimum-Maximum
Age (years)	64.9 (3.1)	64.5 (63.0-66.0)	61.0-73.0
Education (years)	10.3 (2.7)	10.5 (9.0-12.0)	6.0-14.0
Height (cm)	162.0 (9.6)	162.7 (154.0-171.7)	145.8-175.4
Weight (kg)	62.7 (9.8)	59.7 (54.7-72.0)	47.2-75.5
Systolic BP^a^ (mm Hg)	123.5 (13.7)	127.0 (118.0-132.0)	98.0-143.0
Diastolic BP (mm Hg)	75.3 (9.8)	76.0 (68.5-78.0)	62.0-93.0
Pulse (bpm)	71.7 (6.8)	69.5 (65.5-76.5)	64.0-86.0
Body temperature (℃)	36.5 (0.1)	36.5 (36.4-36.5)	36.4-36.6
K-MoCA^b^ score	20.3 (1.1)	20.0 (19.5-21.0)	19.0-22.0

^a^BP: blood pressure.

^b^K-MoCA: Korean version of the Montreal Cognitive Assessment.

The mean age, height, weight, and duration of education of participants were 64.9 (SD 3.1) years, 162.0 (SD 9.6) cm, 62.7 (SD 9.8) kg, and 10.3 (SD 2.7) years (roughly corresponding to a middle to high school level), respectively. At screening, the mean K-MoCA score was 20.3 (SD 1.8), confirming that all participants met the inclusion criteria.

### Recruitment Conditions

All participants reported normal or corrected-to-normal vision sufficient for daily activities. Participants were allowed to wear their habitual corrective devices, including prescription glasses or contact lenses, during all experimental procedures. For near-vision tasks presented on the display, participants with presbyopia were instructed to use their usual near-vision correction to ensure comfortable viewing of on-screen stimuli. At the beginning of each session, participants were asked to confirm that all stimuli were clearly visible at the intended viewing distance, and adjustments (eg, seating position or corrective lenses) were made if necessary. Individuals with known ophthalmologic diseases that could substantially interfere with visual perception or eye movements (eg, advanced cataract, glaucoma, retinal disorders, or severe strabismus) were excluded based on medical history and investigator judgment.

Although no formal ophthalmologic or oculomotor examination was conducted, visual function relevant to task performance was carefully controlled during participant screening and experimental sessions. Adequate visibility and readability of task stimuli were verified prior to task initiation. No participant reported visual discomfort or difficulty performing the task. Color vision was not formally assessed, as task stimuli were designed with high luminance contrast and redundant visual cues, and no task relied solely on color discrimination for correct performance.

### Ethical Considerations

This study was conducted at a single clinical institution and approved by the institutional review board (IRB) of Daejeon University Hospital (IRB number DJUMC-2024-BM-02). The research was carried out at Daejeon University Cheonan Oriental Medicine Hospital until December 2024, following the date of IRB approval.

Participation was entirely voluntary. The principal investigator or research staff explained the study’s objectives, procedures, potential benefits, and possible risks in detail. All participants provided written informed consent before enrollment and were informed that they could withdraw at any time without consequence.

All personal information was kept confidential. Identifying data were replaced with participant codes, and all data were fully anonymized. No personal identifiers were disclosed in any publications arising from the study. Participants received a small participation fee and transportation support.

### Experimental Methodology

Participant selection was based on a preliminary screening process. During screening, interviews, and basic clinical examinations were conducted to assess eligibility, and only those who met the inclusion and exclusion criteria were enrolled. After providing written informed consent, participants’ demographic information (sex, date of birth, height, and weight) was collected, and vital signs (blood pressure, pulse, and body temperature), medical history, and medication use were recorded.

**Figure 1:**
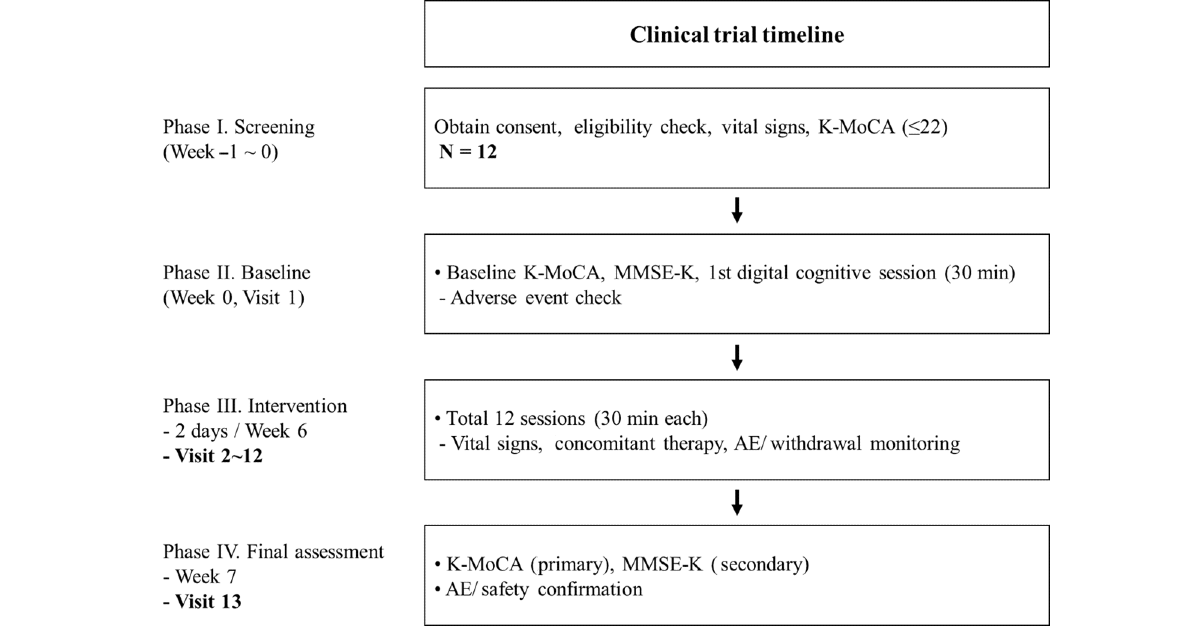
Study enrollment and assessment flow. This schematic illustrates the overall study procedures, including participant screening, enrollment, baseline assessment, the 6-week intervention, and postintervention assessment. Eligible participants were identified through preliminary screening involving interviews and clinical examinations. Participants then completed the EYAS Standard digital eye-movement training program twice weekly for 30 minutes per session over 6 weeks. Cognitive assessments (Korean version of the Montreal Cognitive Assessment [K-MoCA] and Mini-Mental State Examination-Korean version [MMSE-K]) were administered at the first (preintervention) and final (postintervention) visits, and adverse events (AEs) were monitored throughout the study period.

The intervention period lasted 7 weeks, excluding screening. Participants visited the clinical site and performed a digital eye-movement training program, EYAS Standard (Inthetech Co Ltd), under the supervision of medical staff. Training sessions lasted 30 minutes, twice a week, over 6 weeks. At the first (pre) and final (post) visits, cognitive assessments were conducted using the K-MoCA and the Mini-Mental State Examination-Korean version (MMSE-K; Figure 1). A total of 12 participants completed all 12 training sessions without missing any session. The intervention was supervised by trained clinical staff experienced in cognitive assessment and digital therapeutic interventions. Differences between the 2 time points were analyzed to evaluate intervention outcomes. This study was reported in accordance with the TIDieR (Template for Intervention Description and Replication) checklist to enhance the transparency and reproducibility of the intervention (Multimedia Appendix 1).

### Psychological Assessments

Cognitive function was assessed using the K-MoCA and MMSE-K. The K-MoCA is a screening tool developed to detect MCI with high sensitivity [25]. The Korean version has been adapted to reflect cultural and linguistic characteristics. The test comprises 30 points in total, covering orientation (6 points), memory (5 points), visuospatial ability (4 points), executive function (4 points), attention (6 points), and language (5 points) [26]. For participants with ≤6 years of education, 1 additional point is added to adjust for educational effects [27]. The K-MoCA can be administered in a short time while maintaining strong validity for the early detection of cognitive decline [25] and is generally more sensitive than the MMSE in identifying predementia conditions [28,29]. Therefore, the K-MoCA was used as the primary measure of cognitive status in this study.

The MMSE-K is a validated tool for dementia screening in Korea. It is brief (approximately 5-10 minutes) and assesses multiple domains: temporal orientation (5 points), spatial orientation (5 points), registration (3 points), recall (3 points), attention and calculation (5 points), language (7 points), and comprehension/judgment (2 points) [26]. For participants with limited literacy, up to 4 points can be adjusted. The scoring criteria classify ≥24 points as “normal,” 20-23 as “possible dementia,” and ≤19 as “dementia.” The MMSE-K is simple to administer and interpret, making it widely used in community-based studies and clinical screening [30]. In this study, it was used as a complementary measure to confirm overall cognitive status alongside the K-MoCA.

### Eye-Tracking System

Eye movements were recorded using a screen-mounted eye-tracking system (Tobii Eye Tracker 5; Tobii AB), integrated with a Unity-based (Unity Software Inc) digital training environment via the Tobii Software Development Kit (SDK). Participants interacted exclusively through eye movements, without the need for mouse or touch input, with fixation and saccade patterns serving as the primary input signals. The eye tracker operated at a sampling rate of 60 Hz. According to manufacturer specifications, the system provides spatial accuracy within approximately 40×40 degrees of visual angle and a spatial precision of approximately 0.3° root mean square under optimal conditions. The system operates through the following procedures:

1. Calibration: a 3-point or 9-point calibration with the Tobii Eye Tracker aligns the user’s eye position, with results incorporated into gaze-coordinate precision.
2. Data collection: real-time (X, Y) gaze coordinates are acquired via GazePointData (Gazepoint Research Inc) and mapped onto objects within the content to identify the user’s point of fixation.
3. Fixation event detection: when fixation duration exceeds a predefined threshold, an input event is triggered, enabling task responses such as item selection.
4. Saccade analysis: parameters such as amplitude, duration, and velocity are analyzed to evaluate selective attention shifts and visual responsiveness.
5. Binocular analysis: if necessary, a separate analysis of binocular coordinates is performed to detect attentional bias or fusion failure.

Within the training content, these mechanisms allow for automatic responses, such as presenting new tasks, registering answer selections, or advancing to the next stage once the user fixates on a specific target for a sufficient duration. All training contents were implemented within a Unity environment using the Tobii SDK. Textbox 1 summarizes the technical features of the EYAS Standard.

**Technical specifications of EYAS Standard.**

**Tracking device**

- Tobii Eye Tracker 5/Tobii Pro Nano (screen-mounted)

**Integration platform**

- Unity + Tobii Software Development Kit for Unity

**Eye-tracking function**

- Real-time gaze area detection → content interaction and response logging

**Sampling rate**

- 60 Hz

**Spatial accuracy**

- Approximately 40x40 degrees (under optimal conditions)

**Spatial precision**

- Approximately 0.3° root mean square (under optimal conditions)

**Input condition**

- No manual input; fixation duration and saccadic movements function as inputs

**Data format**

- CSV or JSON (including GazePoint, Fixation, Saccade, and time stamp)

### Experimental Setup

Participants were seated at a fixed viewing distance of approximately 60-65 cm from the display. No chin rest or rigid head stabilization device was used, as the Tobii system supports moderate natural head movement. Visual stimuli were presented on a standard monitor with a screen size of approximately 27 inches. A 3-point calibration procedure was performed at the beginning of each session, following the Tobii SDK calibration protocol, to align gaze coordinates with screen space. Calibration quality was visually inspected and repeated when necessary. Participants were instructed to maintain a comfortable and stable posture during task execution.

### Training Program Components: EYAS Standard

The EYAS Standard used in this study is a digital training program integrating cognitive tasks with eye-movement coordination exercises. It was developed for individuals with cognitive impairment or for those at risk of cognitive decline, particularly targeting patients with MCI. The program targets cognitive functions such as memory and executive function through domain-specific digital eye-tracking technology [20]. By combining visual stimuli with visuomotor coordination tasks, the program is designed to stimulate targeted brain regions via eye-movement exercises, thereby facilitating cognitive enhancement.

**Figure 2:**
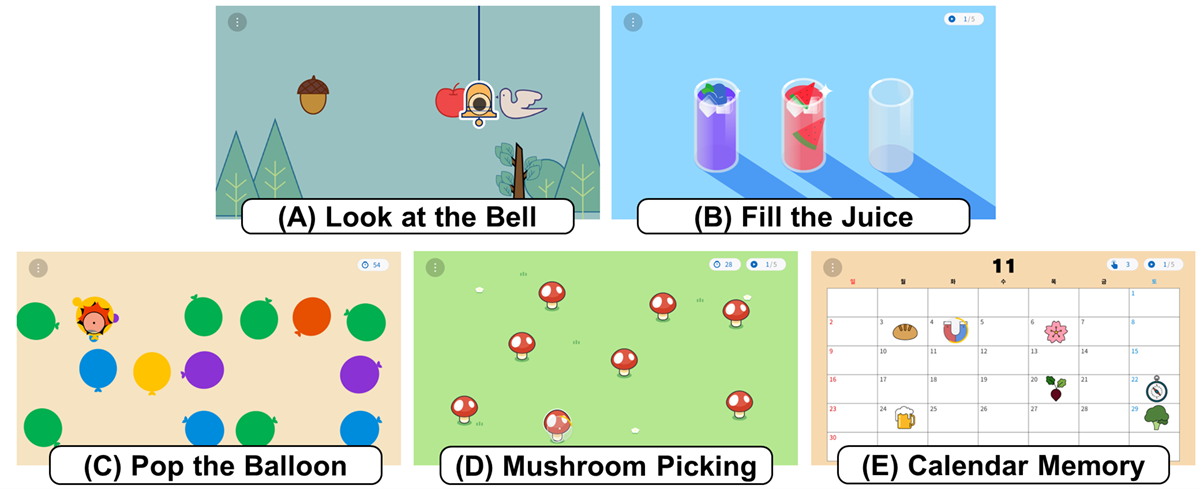
Overview of the EYAS Standard training contents. This figure presents the 5 digital cognitive training contents of the EYAS Standard system: (A) Look at the Bell, assessing alertness through rapid gaze shifts; (B) Fill the Juice, requiring sustained fixation for controlled pouring; (C) Pop the Balloon, measuring reaction speed via immediate fixation responses; (D) Mushroom Picking, evaluating selective attention through visual search of target mushrooms; and (E) Calendar Memory, engaging visuospatial memory with sequential recall of dates. Brief descriptions of each content are provided in Textbox 2.

The EYAS Standard applied in this study comprises 5 distinct training contents (“Look at the Bell,” “Fill the Juice,” “Pop the Balloon,” “Mushroom Picking,” and “Calendar Memory”), each designed to evaluate and stimulate different aspects of cognitive function (Figure 2). The contents target multiple domains, including attention, memory, reaction speed, and visuospatial ability.

A uniform difficulty level protocol was applied across all participants. As the program was administered to patients with MCI, whose eye-tracking performance and oculomotor control are often reduced, the implementation of an adaptive difficulty level was not feasible. Instead, the program was structured with progressively increasing difficulty to facilitate improvement in gaze-tracking ability: sessions 1-4 were set to an easy level, sessions 5-8 to a moderate level, and sessions 9-12 to a high level. The detailed description of each content is provided in Textbox 2.

**Overview of cognitive training contents in EYAS Standard.**

**Look at the Bell**

- Various images are presented on the screen, and participants are instructed to fixate only on the image of a bell. Correct fixations are recorded as fixation duration, which is used to evaluate sustained and selective attention.

**Fill the Juice**

- Several cups with different colors and positions are briefly displayed and then disappear. When an empty cup screen appears, participants respond by moving their gaze (saccades) and fixating on the remembered location of the juice. This task assesses spatial working memory and visuospatial cognitive load.

**Pop the Balloon**

- Balloons of various colors appear and disappear randomly, with a specific color (such as blue) designated as the target. Participants are required to quickly shift their gaze to the target balloon and fixate on it. This task engages color-location associative memory and selective attention.

**Mushroom Picking**

- Participants visually scan the screen to locate mushroom icons and fixate on each target to “pick” it. The task requires deliberate visual search, precise fixation on individual stimuli, and controlled saccadic shifts between targets. This paradigm evaluates selective attention, visual scanning efficiency, and accuracy of eye movement–based interaction.

**Calendar Memory**

- A calendar displaying dates and days of the week is briefly shown and then replaced by a modified version. Participants must recall the changed elements and shift their gaze to the altered position. This task assesses visual memory and spatial recognition.

### Program Collected Data: EYAS Standard

During the execution of each content, participants’ eye-movement data were quantitatively recorded. The primary variables included accuracy, fixation duration, fixation count, saccade amplitude, saccade duration, and saccade velocity. These 6 variables served as core indicators for evaluating participants’ attentional focus, visual search efficiency, oculomotor control, memory processing, and overall cognitive performance. A detailed summary of the collected data metrics is presented in Textbox 3.

**Data metrics collected from EYAS Standard.**

**Accuracy**

- Proportion of correct cognitive responses per session (range 0-1).

**Fixation duration (ms)**

- Average time that the gaze remains fixed on a single target, reflecting sustained attention and processing effort.

**Fixation count**

- Total number of fixations recorded during a task, indicating the frequency of attentional shifts.

**Saccade amplitude**

- Eye movement distance between two consecutive fixations, representing the extent of visual scanning.

**Saccade duration (ms)**

- Time required to complete a saccadic movement, related to visual-motor coordination.

**Saccade velocity (°/s)**

- Saccade amplitude divided by duration (°/s), representing eye movement speed between consecutive fixations.

### Data Analysis

Statistical analyses were conducted to examine changes in cognitive scores and eye-movement metrics following the digital cognitive training program. Given the small sample size (n=12), the normality of the data distributions was assessed using the Shapiro-Wilk test. As the assumption of normality was not satisfied, nonparametric statistical methods were applied. Pre-post differences in global cognitive function were evaluated using the Wilcoxon signed-rank test for K-MoCA and MMSE-K scores. Effect sizes for these comparisons were calculated using Cohen *d* to quantify the magnitude of the intervention effects.

To investigate changes across the 12 sessions based on repeated measurements, correlation analyses were performed between session numbers and eye-movement indicators obtained from each training task. Spearman correlation coefficients were calculated to assess linear associations between training progression and changes in oculomotor metrics, including fixation duration, fixation count, saccade duration, and saccade velocity. Repeated measurements across sessions were considered as longitudinal observations. Although within-subject dependence was not explicitly modeled, the analysis focused on overall trends across sessions.

Additionally, to evaluate changes in eye-movement behavior over the course of the intervention, eye-tracking metrics from the first and final training sessions were compared using the Wilcoxon signed-rank test. This analysis was conducted to identify improvements in oculomotor control, visual search efficiency, and attentional performance following repeated training.

Statistical significance was set at *P*<.05 for all analyses. All statistical analyses were performed using Python (version 3.14; Python Software Foundation).

## Results

### Changes in K-MoCA and MMSE-K Scores After 6 Weeks of Intervention

**Figure 3:**
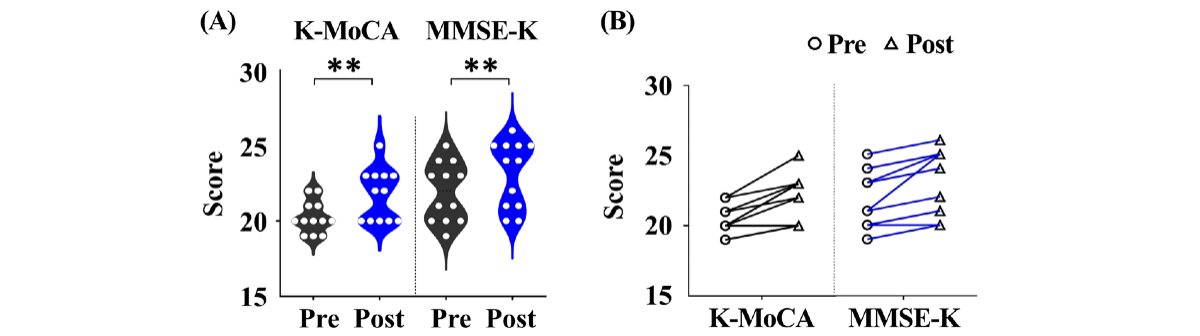
Changes in global cognitive function following digital eye movement–based cognitive training. (A) Violin plots depict pre- and postintervention scores on the Korean version of the Montreal Cognitive Assessment (K-MoCA) and Mini-Mental State Examination-Korean version (MMSE-K). White dots represent individual participants, and the distribution illustrates the score variance across the cohort. Significant improvements were observed in both cognitive measures after the 6-week training program. (B) Paired line plots display individual trajectories from baseline to postintervention for each cognitive test. Nearly all participants demonstrated upward shifts in both K-MoCA and MMSE-K, indicating consistent cognitive gains across the sample. The circle markers denote preintervention scores, and the triangle markers indicate postintervention scores. **P<.01.

Analysis of changes in K-MoCA scores from preintervention to postintervention showed a significant improvement (Figure 3). The mean K-MoCA score increased from 20.3 (SD 1.1) at baseline to 21.8 (SD 1.7) at postintervention, reflecting a significant average gain of 1.5 points (*P*=*.*004; Table 2). The effect size was large (Cohen *d*=1.38; Table 3). These findings suggest that a short-term digital cognitive intervention program can substantially enhance multiple cognitive domains in older adults with MCI, including executive function, memory, and attention.

**Table 2:** Descriptive statistics of effectiveness outcomes.

Characteristic		Preintervention (N=12)	Postintervention (N=12)	Difference (95% CI)		*P* value	
**K-MoCA^a^**				1.5 (0.81-2.19)	.002		
	Mean (SD)	20.3 (1.1)	21.8 (1.7)				
	Median (IQR)	20.0 (19.8-21.0)	22.0 (20.0-23.0)				
	Minimum-Maximum	19.0-22.0	20.0-25.0				
**MMSE-K^b^**				1.3 (0.64-1.86)	.001		
	Mean (SD)	21.9 (2.0)	23.2 (2.2)				
	Median (IQR)	22.0 (20.0-23.3)	24.0 (21.0-25.0)				
	Minimum-Maximum	19.0-25.0	20.0-26.0				

^a^K-MoCA: Korean version of the Montreal Cognitive Assessment.

^b^MMSE-K: Mini-Mental State Examination-Korean version.

**Table 3:** Linear mixed effects model results for effectiveness outcomes.

Outcome and session		EMM^a^ (95% CI)		Difference (95% CI)		*t* test (*df*)		*P* value		Cohen *d* (95% CI)
**K-MoCA^b^**				1.5 (0.8-2.2)		4.78 (11.0)		<.001		1.38 (0.93-2.31)
	Pre	20.3 (19.4-21.2)								
	Post	21.8 (20.9-22.7)								
**MMSE-K^c^**				1.3 (0.6-1.9)		4.49 (11.0)		<.001		1.29 (0.97-3.75)
	Pre	21.9 (20.6-23.3)								
	Post	23.2 (21.8-24.5)								

^a^EMM: estimated marginal means.

^b^K-MoCA: Korean version of the Montreal Cognitive Assessment.

^c^MMSE-K: Mini-Mental State Examination-Korean version.

Similarly, MMSE-K scores significantly increased, from a baseline mean of 21.9 (SD 2.0) to 23.2 (SD 2.2) at postintervention, an average increase of 1.3 points (*P*=*.*002; Table 2). The corresponding effect size was large (Cohen *d*=1.29), indicating that the observed improvements are unlikely to be due to chance and represent meaningful cognitive gains (Table 3). As the MMSE evaluates overall cognitive function, these results highlight that EYAS Standard contributed to domain-specific improvements and to broader cognitive enhancement.

Furthermore, the concept of minimal clinically important difference (MCID) provides additional support for the clinical relevance of these findings. The MCID for the Montreal Cognitive Assessment is generally reported as 1-2 points [31]. A score below 26 on the K-MoCA is commonly used as a screening cutoff for MCI, whereas an MMSE-K score below 24 is generally considered indicative of possible cognitive impairment. In this study, the baseline K-MoCA scores (mean 20.3, SD 1.1; median 20.0, IQR 19.8-21.0) were below the commonly used K-MoCA cutoff, and the MMSE-K scores (mean 21.9, SD 2.0; median 22.0, IQR 20.0-23.3) were also within the range suggestive of cognitive impairment. These score distributions are consistent with the cognitive characteristics of individuals with MCI. Importantly, the observed improvement in K-MoCA scores (mean increase of 1.5 points) falls within the reported range of MCID, suggesting that the change is not only statistically significant but also clinically meaningful. Similarly, the improvement in MMSE-K (1.3 points) approaches thresholds considered clinically relevant in longitudinal cognitive assessments, supporting its potential clinical significance. However, because functional outcomes were not directly assessed in this study, the broader clinical implications of these changes should be interpreted with caution.

### Correlation Between Training Sessions and Eye-Movement Metrics

**Figure 4:**
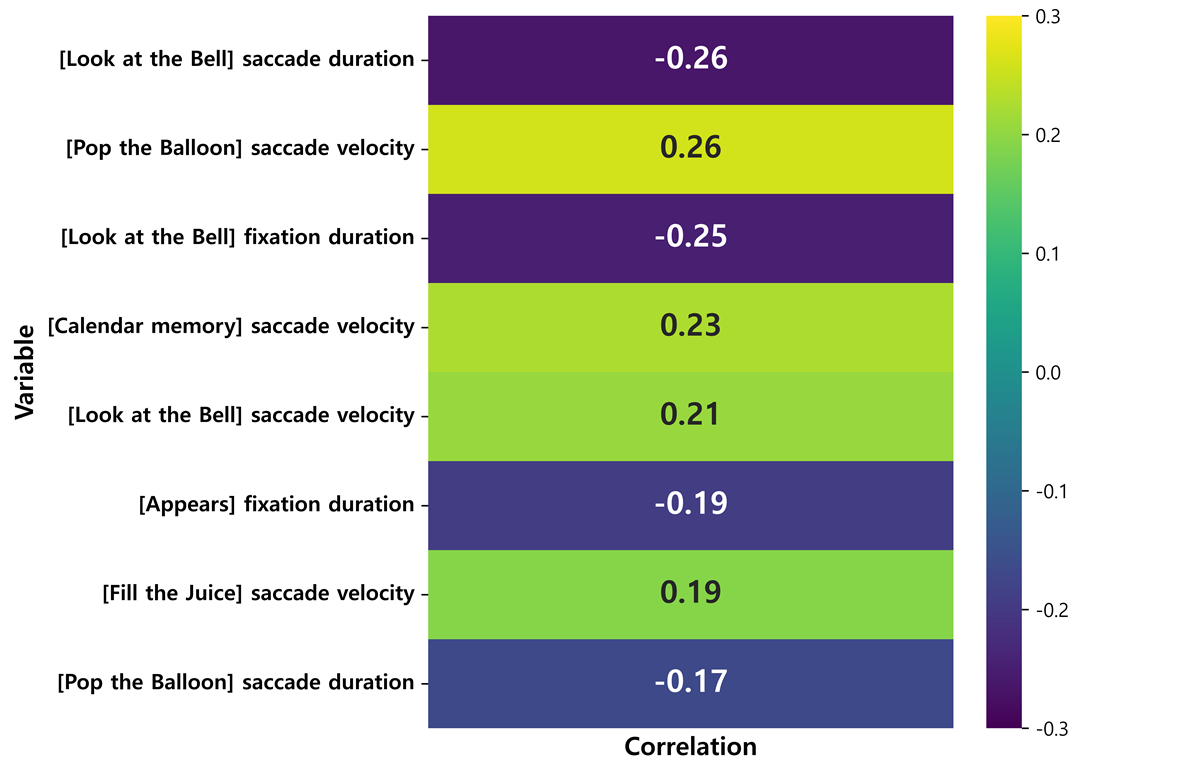
Heatmap of Spearman correlations between variables and training sessions. This figure illustrates the Spearman correlation coefficients between the 12 training sessions and the task variables that showed statistically significant correlations. Positive correlations (green) indicate variables that increase together, whereas negative correlations (blue) represent inverse relationships.

To examine the cumulative effects of digital cognitive training on visuospatial attention, oculomotor performance, and overall cognitive efficiency, a correlation analysis was conducted between training sessions and eye movement–based indicators collected from each task module (Figure 4). The analysis aimed to provide quantitative evidence for cognitive improvement in individuals with MCI by identifying which oculomotor metrics showed progressive enhancement or reduction over the 12 training sessions. Spearman correlation coefficients were calculated between session number and each variable, with statistical significance set at *P*<.05.

Significant correlations were observed across several metrics as the number of training sessions increased (Table 4). Fixation duration demonstrated a significant negative correlation with session number in both the “Look at the Bell” (*r*=–0.248; *P*=*.*003) and “Mushroom Picking” (*r*=–0.194; *P*=*.*02) tasks, indicating that fixation times decreased over repeated sessions. This finding suggests enhanced visual engagement efficiency and faster information processing. In the early sessions, participants required longer fixations to recognize and respond to stimuli, whereas with repeated training, unnecessary fixations were reduced and visual attention was distributed more efficiently.

**Table 4:** Summary of eye-movement measures showing significant correlations with training sessions.

Task	Eye-movement metric	Coefficient, *r*	*P* value
Look at the Bell	Saccade duration	–0.263	.001
Pop the Balloon	Saccade velocity	0.258	.002
Look at the Bell	Fixation duration	–0.248	.003
Calendar Memory	Saccade velocity	0.226	.007
Look at the Bell	Saccade velocity	0.208	.01
Mushroom Picking	Fixation duration	–0.194	.02
Fill the Juice	Saccade velocity	0.192	.02
Pop the Balloon	Saccade duration	–0.167	.045

Regarding saccade metrics, modest changes were observed in selective attention and visuomotor responsiveness. Saccade duration showed significant negative correlations with session number in “Look at the Bell” (*r*=–0.263; *P*=*.*001) and “Pop the Balloon” (*r*=–0.167; *P*=*.*045), suggesting a tendency toward shorter saccadic times as training progressed. This trend may reflect gradual changes in visual search and reaction processes. Conversely, saccade velocity showed significant positive correlations in “Pop the Balloon” (*r*=0.258; *P*=*.*002), “Calendar Memory” (*r*=0.226; *P*=*.*007), “Look at the Bell” (*r*=0.208; *P*=*.*01), and “Fill the Juice” (*r*=0.192; *P*=*.*02), indicating slight increases in eye-movement speed over time. In contrast, fixation count, saccade amplitude, and accuracy did not show significant correlations with session number across any of the tasks, indicating no consistent change in these metrics over time.

Collectively, the decrease in fixation duration and increase in saccade velocity across sessions indicate a consistent shift toward more efficient and automated visual exploration, suggesting that repetitive digital cognitive training progressively refines the gaze patterns of individuals with MCI, reducing unnecessary fixations while improving selective attention and reaction agility. Given the weak correlations, these findings indicate preliminary trends rather than robust effects, suggesting modest improvements in visual search strategies and attentional shifts with continued training.

### Pre-Post Comparison of Eye-Movement Metrics

To assess how digital cognitive training altered participants’ eye-movement behaviors, eye-tracking data from the first and final sessions were directly compared. This pre-post comparison focused on detecting measurable improvements in oculomotor control, visual search speed, and attentional efficiency. Across the 6 eye-movement variables examined for each of the 5 game contents, 3 exhibited significant differences (Table 5).

**Table 5:** Pre-post comparison of eye-movement measures between session 1 and session 12.

Variable	Session 1, mean (SD)	Session 12, mean (SD)	Difference (post – pre)	Wilcoxon statistic	*P* value
Look at the Bell (saccade duration)	69.025 (18.101)	47.308 (18.272)	–21.717	6.0	.007
Calendar Memory (saccade velocity)	307.600 (141.995)	422.142 (134.135)	114.542	4.0	.01
Fill the Juice (saccade velocity)	298.342 (127.879)	393.058 (132.153)	94.717	9.0	.02

Among these, saccade duration in the “Look at the Bell” content significantly decreased from a mean of 69.03 (SD 18.10) ms at session 1 to 47.31 (SD 18.27) ms at session 12, representing a reduction of 21.72 ms (W=6.0; *P*=*.*007). This decline may indicate faster gaze transitions between target stimuli, suggesting more efficient visual information processing with continued training. This result may reflect improvements in visual search efficiency and oculomotor responsiveness, potentially driven by increased automaticity in eye-movement control.

In the “Calendar Memory” task, saccade velocity significantly increased (W=4.0; *P*=*.*01), rising from a mean of 307.60 (SD 141.99) °/s at session 1 to 422.14 (SD 134.14) °/s at session 12, a difference of 114.54 °/s. This finding indicates faster processing of visual cues and more efficient retrieval of relevant spatial information, reflecting improvements in information retrieval speed and visuospatial coordination.

Similarly, in the “Fill the Juice” task, saccade velocity significantly increased from a mean of 298.34 (SD 127.88) °/s to 393.06 (SD 132.15) °/s, with a difference of 94.72 °/s (W=9.0; *P*=*.*02). This acceleration in eye-movement speed indicates enhanced visuomotor integration and improved attentional shifting. Given that this task requires recalling spatial locations and executing precise gaze shifts, the result suggests that spatial working memory and selective attention improved over repeated sessions.

Overall, these pre-post comparisons provide clear evidence that digital cognitive training produced measurable improvements in oculomotor performance. The combination of reduced saccade duration and increased saccade velocity demonstrates that participants’ visual exploration became more efficient and automatic, signifying enhanced attentional control, visuospatial awareness, and cognitive processing speed following repeated digital cognitive training.

## Discussion

### Principal Findings

We conducted a 12-session digital cognitive training program for individuals with MCI and examined changes in visuospatial attention, visual search efficiency, and overall cognitive performance using eye-movement indicators collected throughout the sessions. Unlike previous cognitive training studies that mainly relied on neuropsychological test scores or behavioral outcomes, we used continuous and objective eye-tracking data to capture subtle patterns of cognitive change. Eye movements are closely linked to how individuals allocate attention, update working memory, and exert executive control, making them a sensitive marker of cognitive and attentional dynamics during training [32]. Therefore, this study provides a practical framework for assessing the cognitive effects of digital training interventions. Importantly, the magnitude of cognitive improvement observed in this study aligns with established thresholds for clinically meaningful change, suggesting that the intervention may have practical relevance beyond statistical significance.

Correlation analyses between training sessions and eye-movement measures showed gradual improvements in visual attention and search efficiency. In particular, fixation duration demonstrated significant negative correlations with session number, indicating that repeated training reduced fixation time and improved processing efficiency. Saccade duration also decreased across sessions, suggesting faster visuomotor responses and more efficient attentional shifts. In contrast, saccade velocity increased with training, reflecting quicker transitions of gaze and reduced reaction delays. These findings suggest that digital cognitive training contributes to improvements in visual processing speed and attentional control [33,34]. Individuals with MCI often exhibit slow reactions, impaired attention, and inefficient visual scanning patterns [35,36]. The progressive improvements observed in this study suggest that such difficulties can be partially reduced through repeated and structured training. Notably, the reduction in fixation duration may reflect both faster visual processing and more stable sustained attention [37,38], potentially associated with strengthened functional connections within the frontoparietal attention network [39,40]. Overall, the significant correlations between training sessions and oculomotor indicators suggest that digital cognitive training exerts cumulative effects on attention, visual processing, and response control. However, given the weak correlation magnitudes, these findings should be interpreted as preliminary trends rather than as robust effects.

Results from Wilcoxon signed-rank tests comparing session 1 and 12 clearly showed the cumulative effects of the training. A significant reduction in saccade duration indicates faster gaze transitions between visual targets, reflecting improved efficiency in visual search and oculomotor responsiveness [41,42]. In addition, the significant increase in saccade velocity suggests that participants became faster in processing visual information and executing goal-directed eye movements over time [43]. For individuals with MCI, who typically exhibit slowed processing speed and delayed visuomotor responses, such changes may reflect partial improvement in attentional control and cognitive processing efficiency [34,36]. Overall, these findings indicate that digital cognitive training contributes to improved oculomotor efficiency, particularly in terms of processing speed and attentional dynamics, rather than to broad changes in visual scanning patterns.

The training program implemented in this study consisted of both cognitive tasks and eye-movement coordination exercises, designed to simultaneously engage cognitive processing and oculomotor control. However, in analyses incorporating eye-tracking metrics, no significant effects on task accuracy were observed across any content. This relatively weak effect suggests that the observed cognitive improvements in individuals with MCI may not be primarily driven by task-specific accuracy. The visual-cognitive tasks repeatedly required discrimination, prediction, and response regulation, which likely promoted coordination between the frontoparietal attention network and the visual cortex [44]. Consequently, participants showed more consistent visual search behavior and more stable responses, with fewer selection errors and faster reaction times. Instead, the changes may reflect enhancements in attentional dynamics mediated through eye-movement processes. Therefore, the cognitive improvements identified in this study may have been partially mediated by improvements in oculomotor control rather than by direct enhancement of core cognitive functions. Altogether, these findings suggest that digital cognitive training may strengthen the integration between visual and attentional systems, potentially supporting improvements in cognitive function and functional efficiency.

The digital cognitive training program used in this study demonstrated several practical advantages over traditional paper-based or repetitive exercises. First, the integration of eye-tracking enabled objective and continuous quantification of oculomotor and attentional behaviors during task performance. This approach allowed the detection of subtle, process-level changes that are not easily captured by conventional outcome measures. Second, automated eye-movement tracking enabled continuous and objective monitoring of changes in attentional and oculomotor behavior across sessions, providing a more sensitive assessment of within-subject changes than conventional pre-post evaluations. Third, the digital format was noninvasive and easy to use, reducing physical and mental burden for older adults and individuals with MCI, and facilitating consistent self-guided training outside clinical settings. In summary, the training protocol used in this study demonstrates the potential of digital cognitive programs as effective, accessible, and scalable tools for early cognitive intervention and maintenance in at-risk populations.

### Limitations

This study has some limitations. First, the single-group pre-post design and small sample size (n=12) limit causal inference and generalizability. Because this pilot study included a relatively small sample, the large effect sizes observed may be inflated and should therefore be interpreted cautiously as exploratory estimates. Second, the mean age of participants in this study was relatively young (mean 64.9, SD 3.1 years), which is lower than the average age typically reported in many MCI cohorts. Therefore, the findings may have limited generalizability to older populations with MCI, who may exhibit different cognitive trajectories and responses to digital cognitive training. Third, the absence of a control group makes it difficult to determine whether the observed improvements reflect the specific effects of the digital cognitive training or are attributable to practice and retest effects associated with repeated assessments and task exposure. Fourth, multiple eye-movement variables were analyzed without applying statistical correction for multiple comparisons, which may increase the risk of false-positive findings. Fifth, because eye-movement metrics were both trained and evaluated within the same task environment, the observed improvements may partly reflect task-specific learning or increased familiarity with the task structure rather than generalized cognitive transfer. Furthermore, session-to-session variability was not explicitly analyzed, limiting the ability to assess the stability and consistency of performance changes across training sessions. Therefore, the findings of this pilot study should be interpreted cautiously as preliminary and exploratory. Future studies should incorporate appropriate control conditions, larger samples, and independent outcome measures to better distinguish generalized cognitive improvement from task-specific learning and to validate the robustness of the observed effects.

### Future Work

Future research should replicate these findings through large-scale randomized controlled trials with more diverse participant groups to improve generalizability and confirm the robustness of the training effects. Expanding the participant pool will help identify broader applicability and strengthen the external validity of digital cognitive interventions. In addition, in future studies, participants should be stratified by cognitive impairment level, or tailored training modules should be applied to determine which subgroups benefit most, advancing precision-based cognitive rehabilitation for MCI. Finally, integrating adaptive algorithms that adjust task difficulty in real time based on eye-tracking performance may further enhance training efficiency and engagement by maintaining optimal challenge levels and reducing fatigue.

### Conclusion

This study reports that a digital cognitive training program that integrates eye movement–based tasks improved visuospatial attention and oculomotor efficiency in individuals with MCI, as evidenced by significant changes in eye-movement parameters. The progressive optimization of fixation and saccade patterns suggests enhanced attentional control and information processing efficiency through repeated training. These findings highlight the value of eye movement–based metrics as sensitive and quantifiable indicators of cognitive processing efficiency during digital training. Moreover, the integration of real-time eye-tracking data demonstrates the potential of digital cognitive platforms to deliver personalized, adaptive interventions that effectively enhance attention and information-processing abilities in individuals with cognitive impairment. Collectively, this work provides preliminary evidence that technology-assisted cognitive training may represent a scalable, noninvasive tool for promoting cognitive resilience in aging and early-stage neurocognitive disorders.

## Appendix

Multimedia Appendix 1 The TIDieR (Template for Intervention Description and Replication) checklist.

Multimedia Appendix 2 ChatGPT records.

## Abbreviations

IRB: institutional review board
K-MoCA: Korean version of the Montreal Cognitive Assessment
MCI: mild cognitive impairment
MCID: minimal clinically important difference
MMSE-K: Mini-Mental State Examination-Korean version
SDK: Software Development Kit
TIDieR: Template for Intervention Description and Replication

## Data Availability

The datasets generated and analyzed in this study are restricted to the scope originally approved by the institutional review board (IRB). In addition, even for deidentified datasets with all personal information removed, additional IRB review and approval would be required to assess the feasibility of data sharing; therefore, the data cannot be shared at this stage.
