# Supplementary material for: Effects of an Eye-Tracking Digital Serious Game on Cognitive Function in Mild Cognitive Impairment: Pilot Intervention Study
Source: JMIR Form Res. 2026 May 19;10:e88924. doi: 10.2196/88924 (PMC13231112; doi:10.2196/88924)
Supplement: Multimedia Appendix 1 [file formative_v10i1e88924_app1.docx]

**
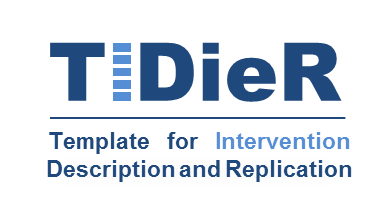
The TIDieR (Template for Intervention Description and Replication) Checklist*:**

Information to include when describing an intervention and the location of the information

| **Item number** | **Item** | **Where located **** | |
| --- | --- | --- | --- |
|  |  | Primary paper  (page or appendix  number) | Other ^†^ (details) |
|  | **BRIEF NAME** |  |  |
| **1.** | EYAS Standard digital eye-movement training program | __Pages 8-10__ | ______________ |
|  | **WHY** |  |  |
| **2.** | The intervention is based on the premise that eye movements are closely linked to cognitive processes such as attention, working memory, and executive control, and that training these oculomotor functions through digital tasks can enhance cognitive performance. By integrating eye-movement–based tasks into a digital training environment, the program aims to improve visuospatial attention, visual processing efficiency, and overall cognitive function in individuals with mild cognitive impairment. | Introduction + Methods + Discussion | _____________ |
|  | **WHAT** |  |  |
| **3.** | The intervention utilized the EYAS Standard digital cognitive training program, which comprises five eye-movement–based task modules (“Look at the Bell,” “Fill the Juice,” “Pop the Balloon,” “Mushroom Picking,” and “Calendar Memory”). Eye movements were recorded using a screen-mounted eye-tracking device (Tobii Eye Tracker 5), integrated with a Unity-based platform via the Tobii SDK. The system collected gaze data including fixation and saccade metrics (e.g., fixation duration, fixation count, saccade amplitude, duration, and velocity) for real-time interaction and performance monitoring. | __Pages 8-12__ | _____________ |
| **4.** | Participants attended supervised training sessions in which they performed the EYAS Standard digital eye-movement–based cognitive training program. Each session began with eye-tracker calibration to align gaze coordinates, followed by task execution using gaze-based interaction without manual input. Participants completed five task modules designed to engage attention, memory, and visuospatial processing through fixation and saccadic eye movements. The intervention consisted of 30-minute sessions conducted twice weekly over 6 weeks. Cognitive assessments (K-MoCA and MMSE-K) were administered at baseline and after completion of the intervention to evaluate changes in cognitive function. | __Pages 6-7__ | _____________ |
|  | **WHO PROVIDED** |  |  |
| **5.** | The research was carried out at Daejeon University Cheonan Oriental Medicine Hospital until December 2024, following the date of IRB approval. | ___Page 6_ __ | _____________ |
|  | **HOW** |  |  |
| **6.** | The intervention was delivered in a face-to-face, on-site clinical setting using a screen-based digital platform integrated with an eye-tracking system. Participants interacted individually with the training program through gaze-based input without manual devices, under the supervision of medical staff. | __Pages 6-7__ | _____________ |
|  | **WHERE** |  |  |
| **7.** | The intervention was conducted at a clinical research site, where participants performed the training using a screen-based digital system integrated with an eye-tracking device. Participants were seated at a fixed viewing distance (approximately 60–65 cm) from a monitor in a controlled indoor environment without head stabilization, allowing natural head movement. The setup included a screen-mounted eye tracker and standard display equipment to support gaze-based interaction. | ___Page 9_ __ | _____________ |
|  | **WHEN and HOW MUCH** |  |  |
| **8.** | The intervention consisted of 12 sessions delivered over a 6-week period. Participants attended training sessions twice per week, with each session lasting approximately 30 minutes. The training followed a progressively increasing difficulty structure, with sessions 1–4 at an easy level, sessions 5–8 at a moderate level, and sessions 9–12 at a high level. | ___Page 10_ __ | _____________ |
|  | **TAILORING** |  |  |
| **9.** | A uniform difficulty level protocol was applied across all participants. As the program was administered to patients with MCI, whose eye-tracking performance and oculomotor control are often reduced, the implementation of adaptive difficulty level was not feasible. Instead, the program was structured with a progressively increasing difficulty to facilitate improvement in gaze-tracking ability: sessions 1–4 were set to an easy level, sessions 5–8 to a moderate level, and sessions 9–12 to a high level. The detailed description of each content is provided in Table 3. | ___ Page10___ | _____________ |
|  | **MODIFICATIONS** |  |  |
| **10.^ǂ^** | No changes during the study | _____N/A_____ | _____________ |
|  | **HOW WELL** |  |  |
| **11.** | Planned: There was no separate strategy, and the participation rate was verified by tallying the number of times each participant participated. | _____N/A_____ | _____________ |
| **12.^ǂ^** | Actual: A total of 12 participants completed all 12 sessions of training without missing. | ___Page 7_ __ | _____________ |

** **Authors** - use N/A if an item is not applicable for the intervention being described. **Reviewers** – use ‘?’ if information about the element is not reported/not sufficiently reported.

† If the information is not provided in the primary paper, give details of where this information is available. This may include locations such as a published protocol or other published papers (provide citation details) or a website (provide the URL).

ǂ If completing the TIDieR checklist for a protocol, these items are not relevant to the protocol and cannot be described until the study is complete.

* We strongly recommend using this checklist in conjunction with the TIDieR guide (see *BMJ* 2014;348:g1687) which contains an explanation and elaboration for each item.

* The focus of TIDieR is on reporting details of the intervention elements (and where relevant, comparison elements) of a study. Other elements and methodological features of studies are covered by other reporting statements and checklists and have not been duplicated as part of the TIDieR checklist. When a **randomised trial** is being reported, the TIDieR checklist should be used in conjunction with the CONSORT statement (see [www.consort-statement.org](http://www.consort-statement.org)) as an extension of **Item 5 of the CONSORT 2010 Statement.** When a **clinical trial** **protocol** is being reported, the TIDieR checklist should be used in conjunction with the SPIRIT statement as an extension of **Item 11 of the SPIRIT 2013 Statement** (see [www.spirit-statement.org](http://www.spirit-statement.org)). For alternate study designs, TIDieR can be used in conjunction with the appropriate checklist for that study design (see [www.equator-network.org](http://www.equator-network.org)).
