# Supplementary material for: Effects of an Eye-Tracking Digital Serious Game on Cognitive Function in Mild Cognitive Impairment: Pilot Intervention Study
Source: JMIR Form Res. 2026 May 19;10:e88924. doi: 10.2196/88924 (PMC13231112; doi:10.2196/88924)

**Effects of an Eye-Tracking Digital Serious Game on Cognitive Function in Mild Cognitive Impairment: A Pilot Intervention Study**

**Sang Woo Lee^1, 3^, MS | Jun-Su Kim^2, 3^, MS | Seung-Jae Kim^2, 3^, MS | Seung-Ho Choun^1^, | Jeong-Heon Song^2,*^, PhD**

^1^ InTheTech Inc., 96, Hyeoksin-daero, Daegu, Republic of Korea, 41065

^2^AI-based Neurodevelopmental Diseases Digital Therapeutics Group, Korea Brain Research Institute (KBRI), 61, Cheomdan-ro, Daegu, Republic of Korea

^3^These authors contributed equally to this work.

* Corresponding author:

**Jeong-Heon Song**: AI-based Neurodevelopmental Diseases Digital Therapeutics Group, Korea Brain Research Institute (KBRI), 61, Cheomdan-ro, Daegu, 41062, Republic of Korea, Tel: 82-53-980-5670, Fax: 85-53-980-8309; jhsong@kbri.re.kr

## Disclosure of ChatGPT Use in Manuscript Preparation

This appendix provides a record of interactions with ChatGPT used exclusively for English language editing during the manuscript preparation process. These interactions were limited to improving clarity, grammar, and readability. No generative AI tools were used for literature review, data analysis, interpretation of results, or generation of scientific content. All AI-assisted outputs were critically reviewed and revised by the authors, who take full responsibility for the final manuscript.

## Complete Generative AI Disclosure

The GAI tool used was: ChatGPT-5.2.

Responsibility for the final manuscript lies entirely
with the authors.
GAI tools are not listed as authors and do not bear
responsibility for the final outcomes.

Declaration submitted by: Jeong-Heon Song

Additional note: We used ChatGPT-5.2 as a generative artificial
intelligence (GAI) tool to assist with English language editing during
manuscript preparation. The use of GAI was limited to language editing, and the manuscript was subsequently revised by a professional editing service.

## ChatGPT Interaction Records

Screenshot of ChatGPT interactions demonstrating the use of AI exclusively for English language editing purposes. All scientific content was generated and verified by the authors.


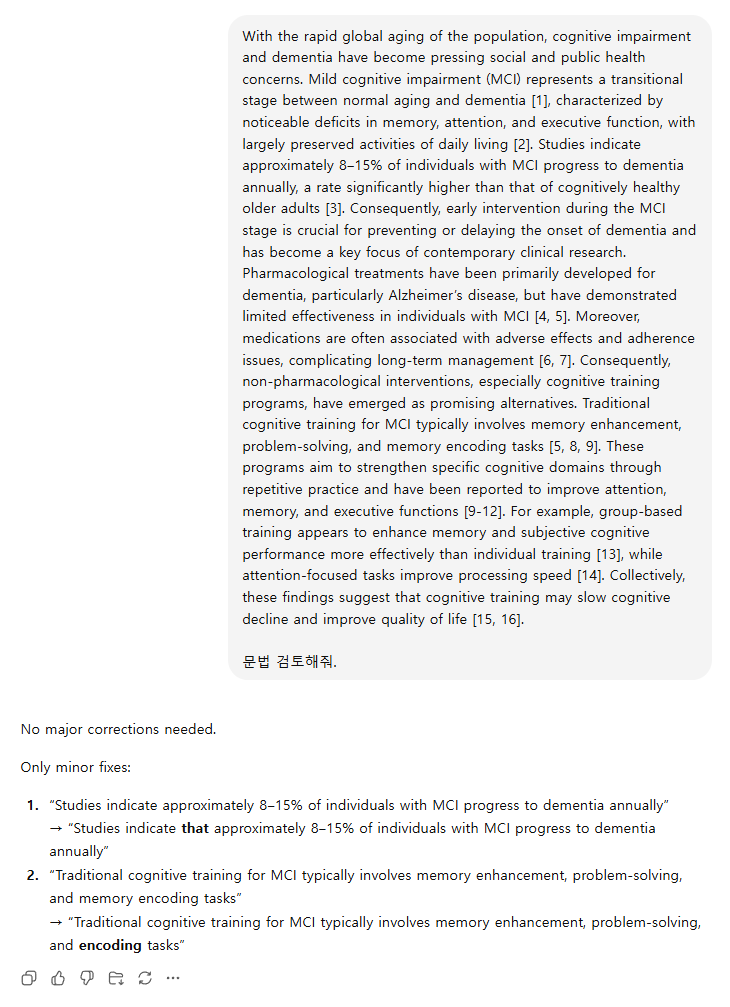


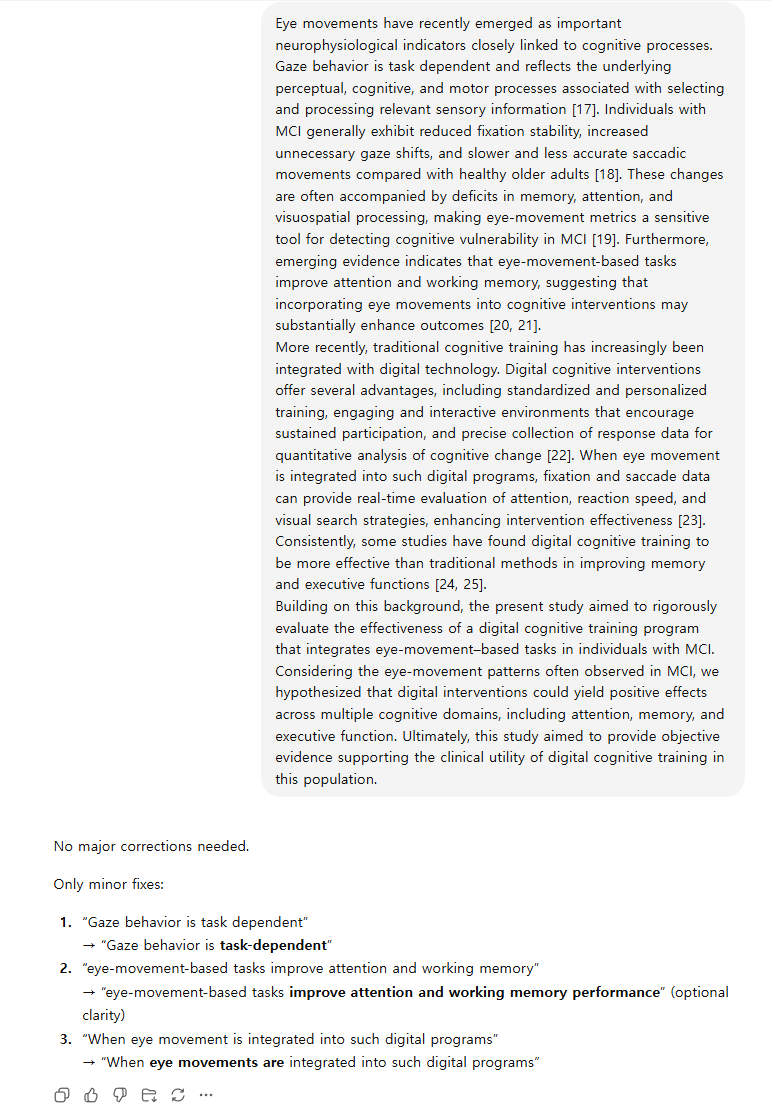


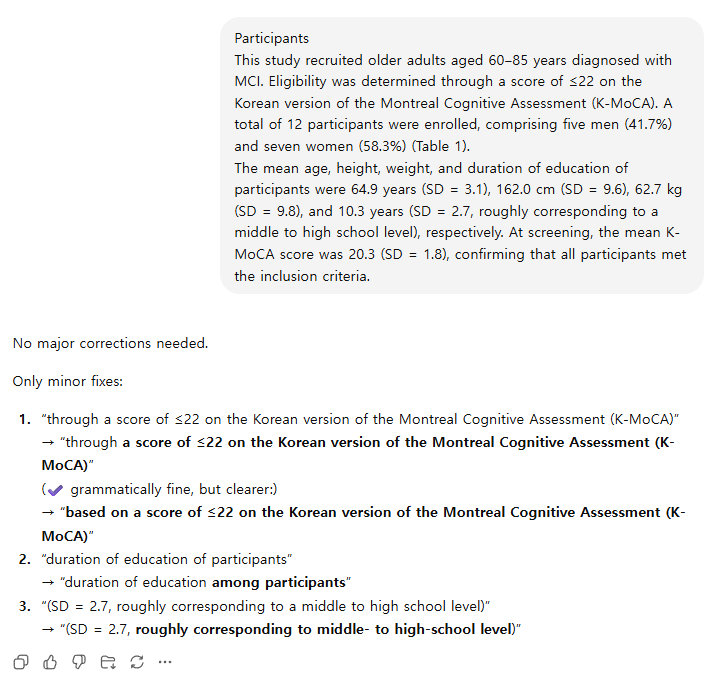


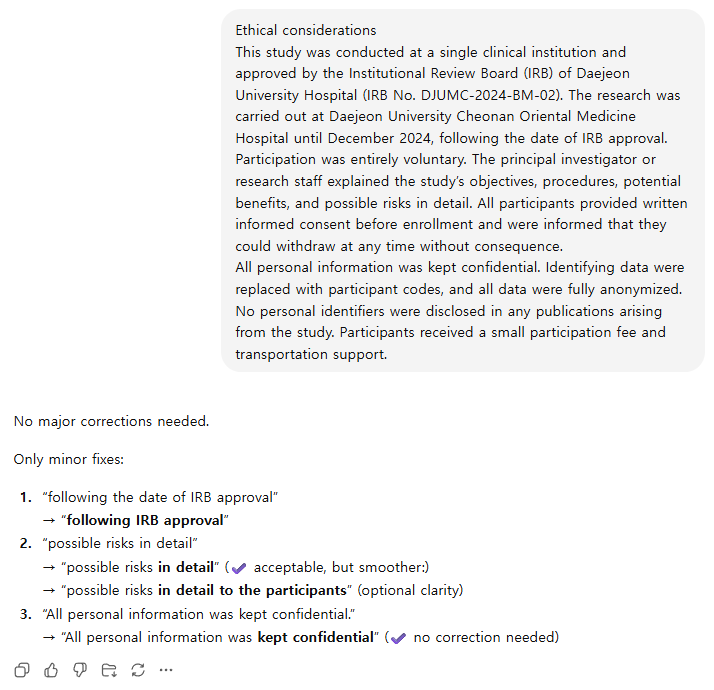


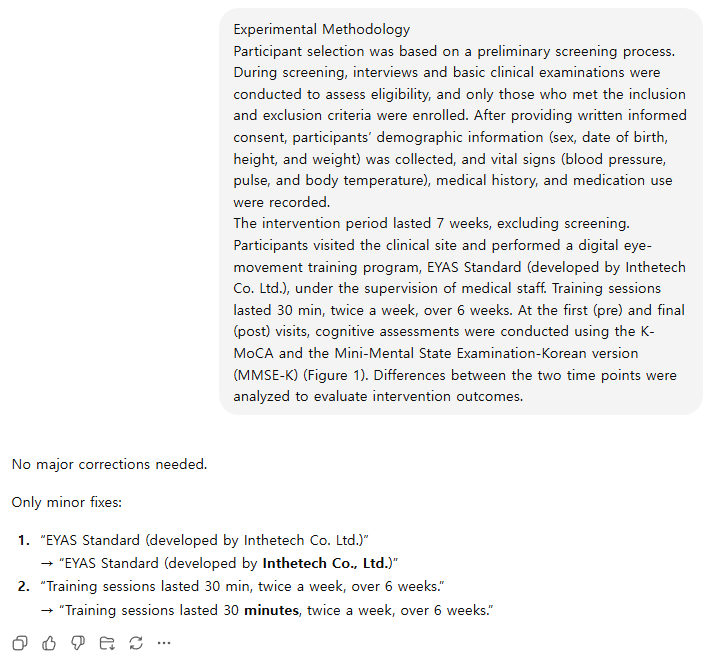


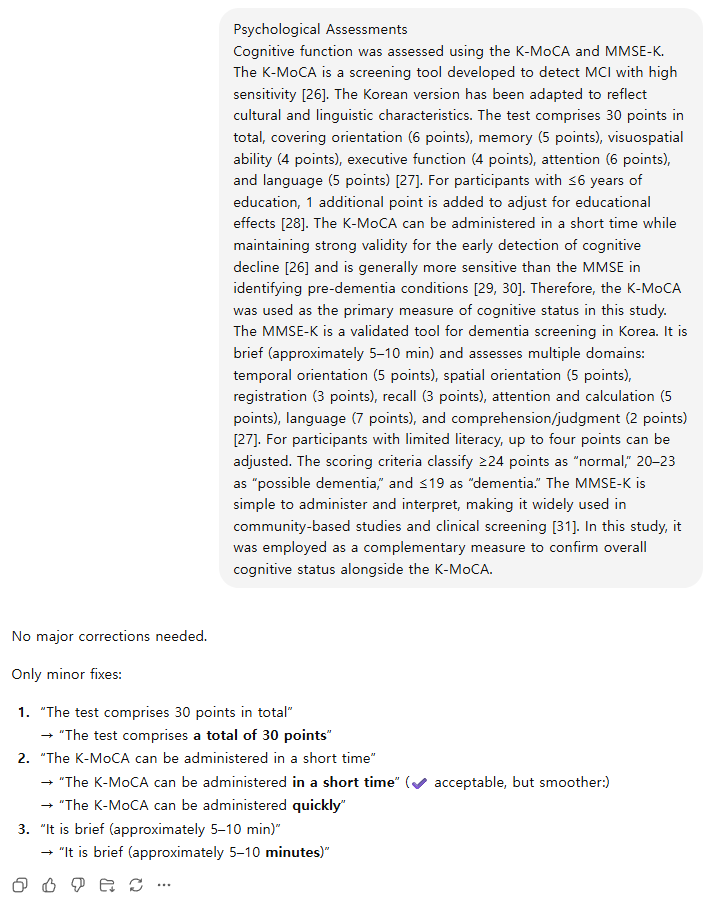


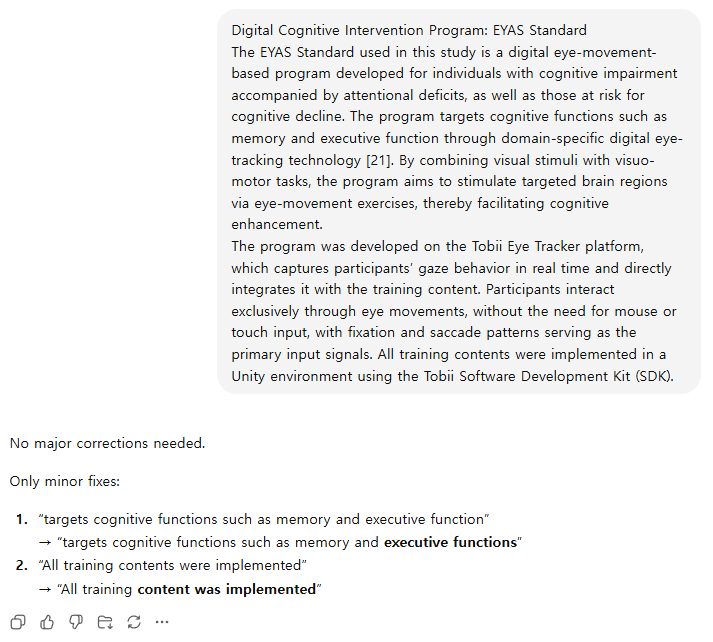


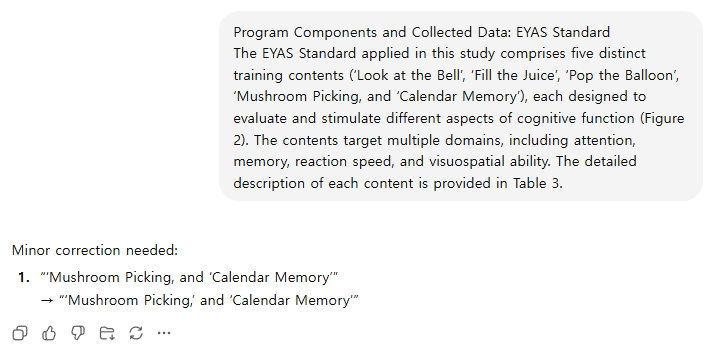


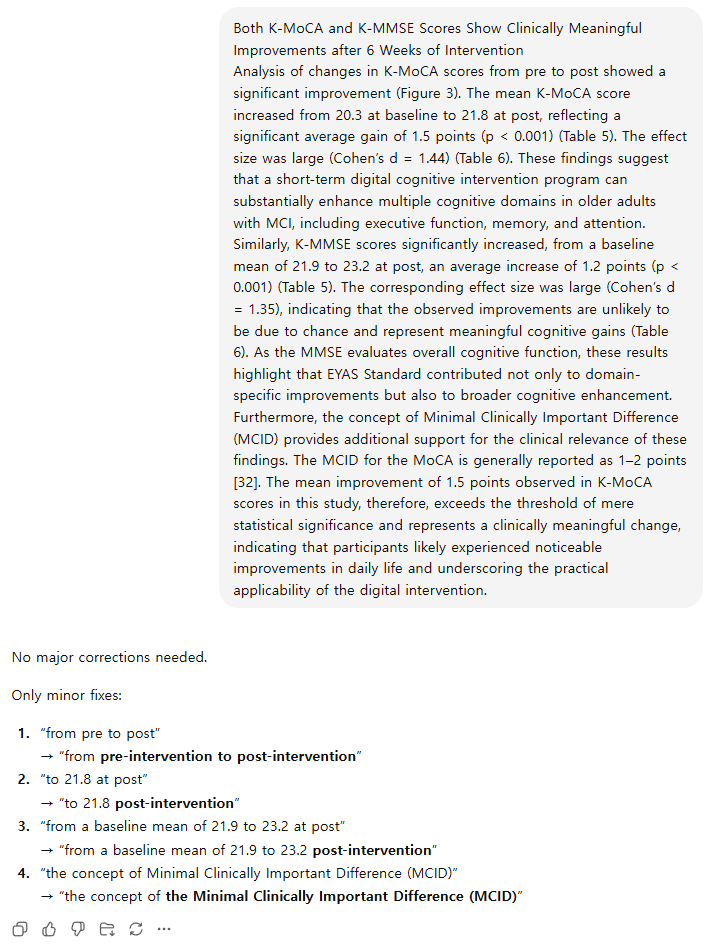


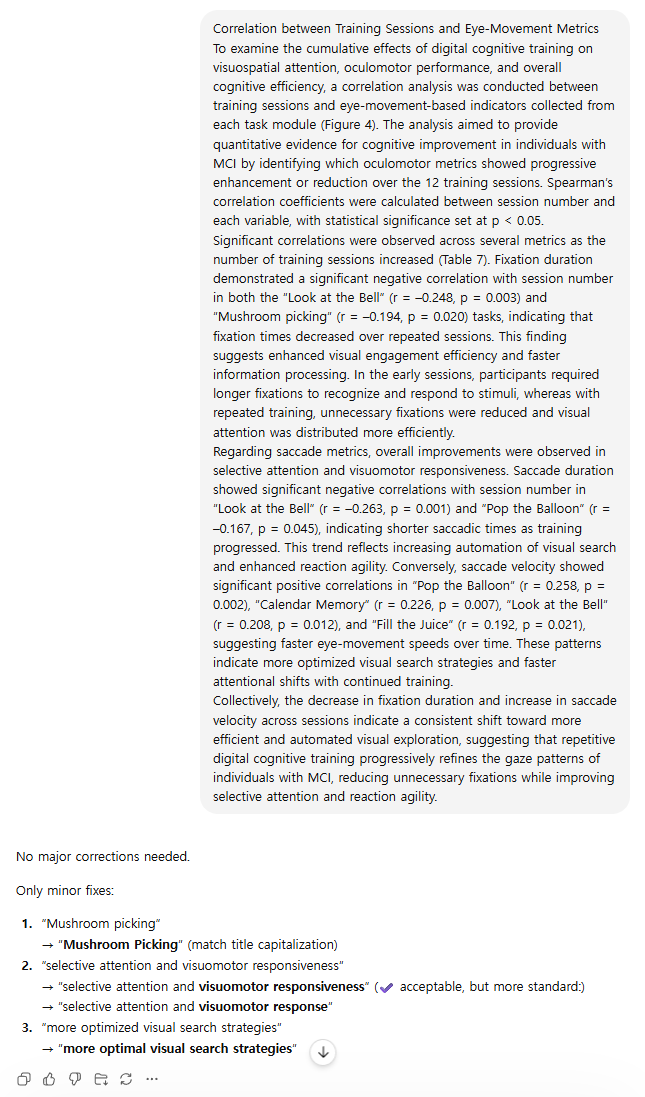


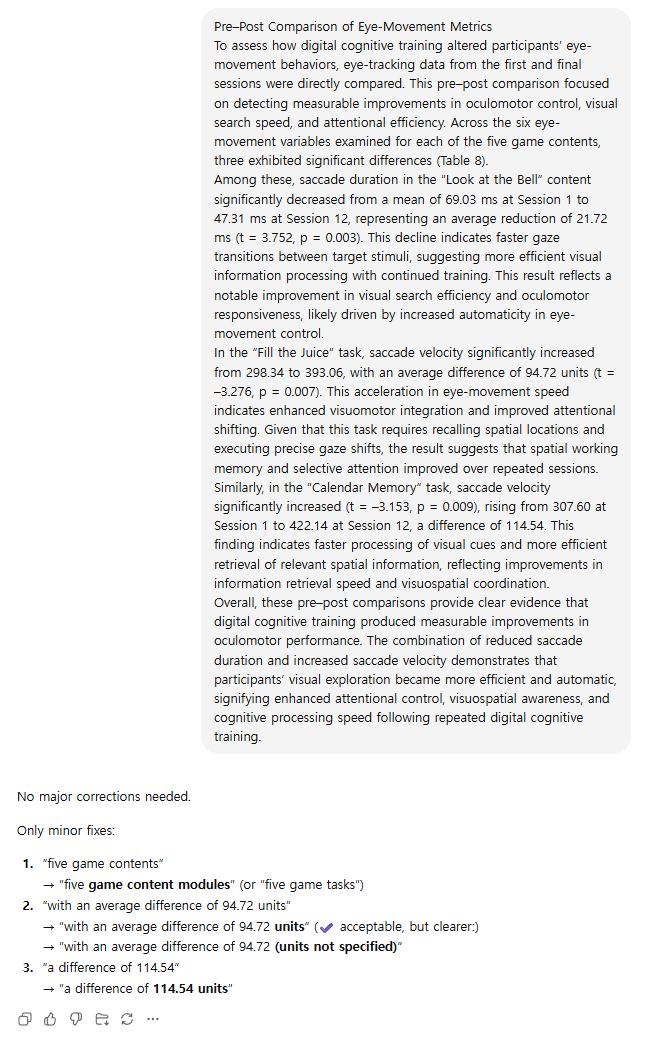


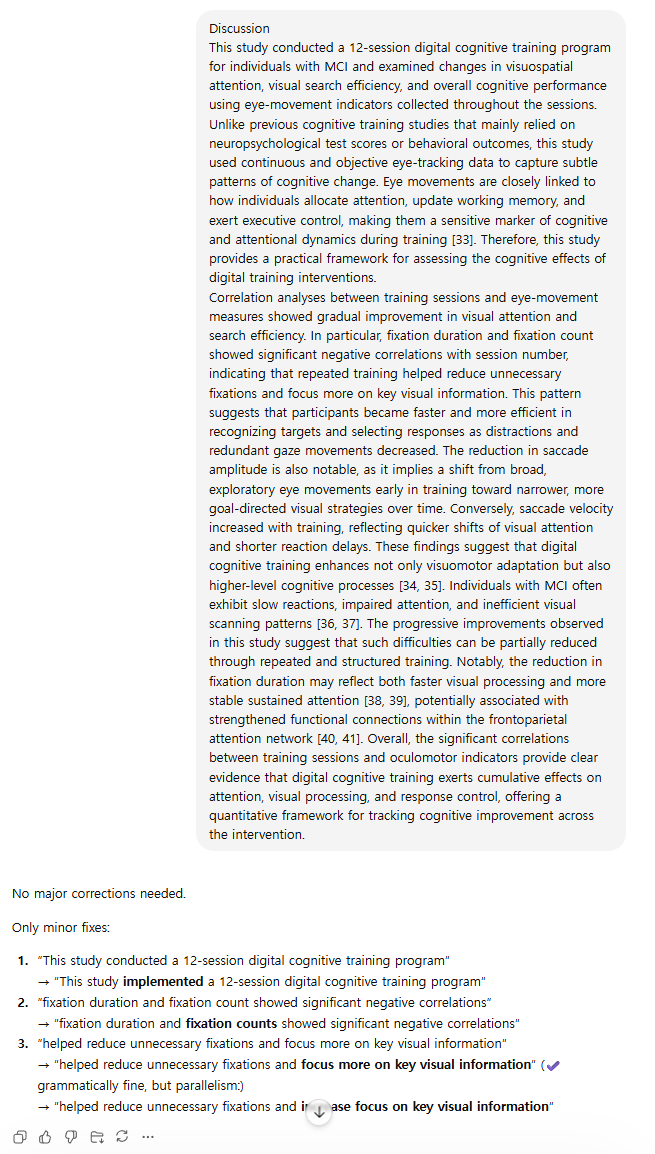


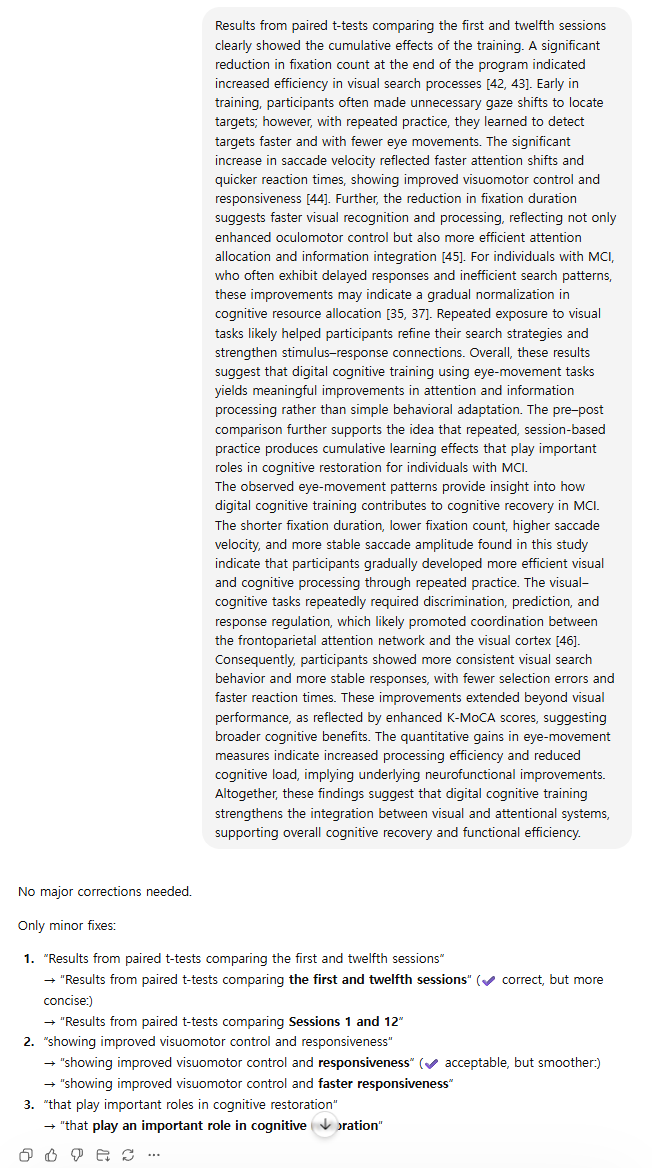


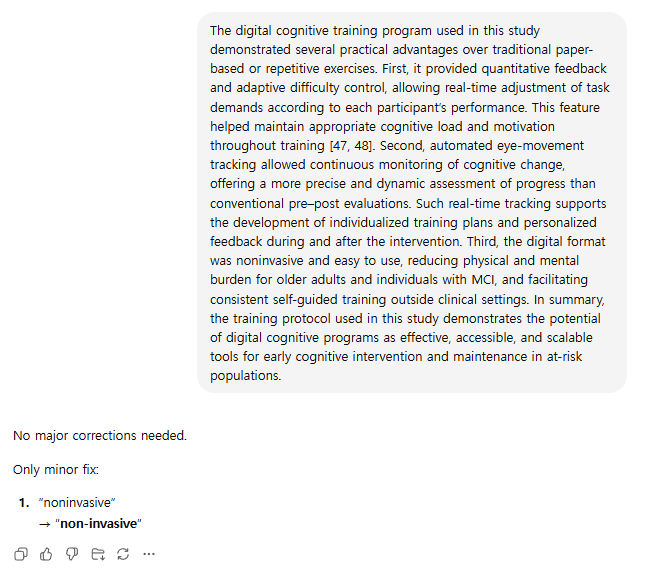


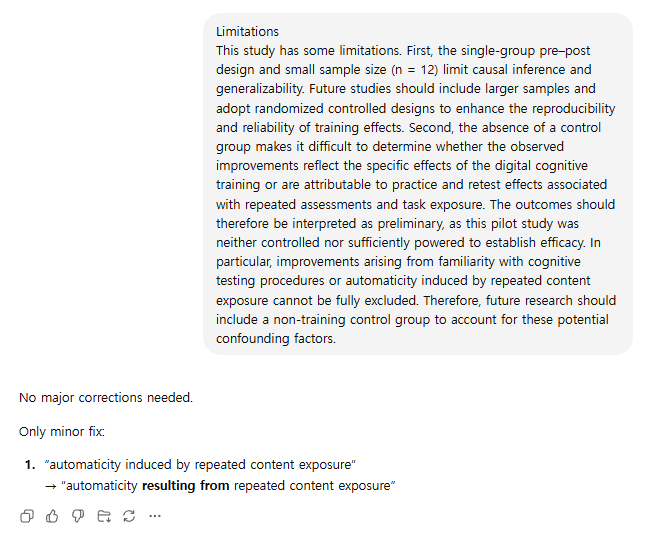


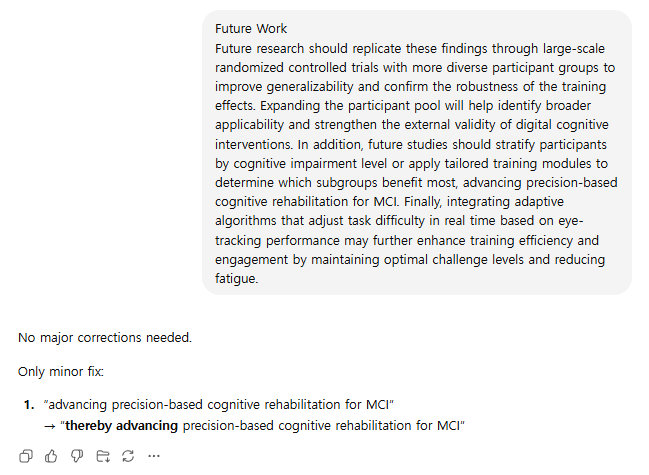


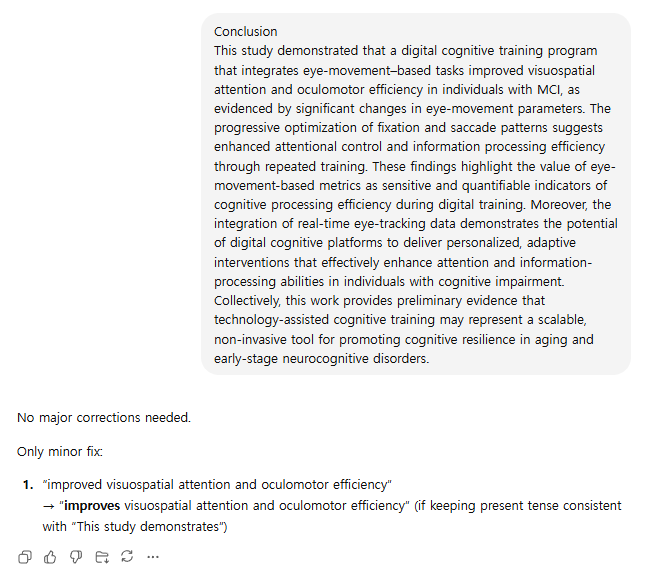


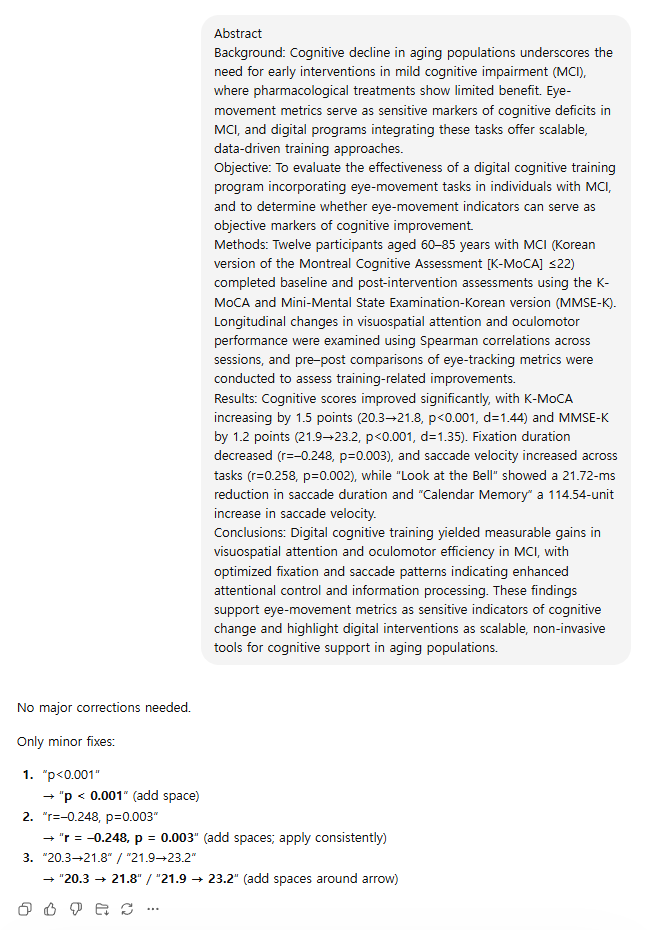

Supplement: Multimedia Appendix 2 [file formative_v10i1e88924_app2.docx]
